# Transgenic Sugarcane with a *cry1Ac* Gene Exhibited Better Phenotypic Traits and Enhanced Resistance against Sugarcane Borer

**DOI:** 10.1371/journal.pone.0153929

**Published:** 2016-04-19

**Authors:** Shiwu Gao, Yingying Yang, Chunfeng Wang, Jinlong Guo, Dinggang Zhou, Qibin Wu, Yachun Su, Liping Xu, Youxiong Que

**Affiliations:** Key Laboratory of Sugarcane Biology and Genetic Breeding, Ministry of Agriculture, Fujian Agriculture and Forestry University, Fuzhou, 350002, Fujian, China; Instituto de Biología Molecular y Celular de Plantas (IBMCP), SPAIN

## Abstract

We developed sugarcane plants with improved resistance to the sugarcane borer, *Diatraea saccharalis* (F). An expression vector pGcry1Ac0229, harboring the *cry1Ac* gene and the selectable marker gene, *bar*, was constructed. This construct was introduced into the sugarcane cultivar FN15 by particle bombardment. Transformed plantlets were identified after selection with Phosphinothricin (PPT) and Basta. Plantlets were then screened by PCR based on the presence of *cry1Ac* and 14 *cry1Ac* positive plantlets were identified. Real-time quantitative PCR (RT-qPCR) revealed that the copy number of *cry1Ac* gene in the transgenic lines varied from 1 to 148. ELISA analysis showed that Cry1Ac protein levels in 7 transgenic lines ranged from 0.85 μg/FWg to 70.92 μg/FWg in leaves and 0.04 μg/FWg to 7.22 μg/FWg in stems, and negatively correlated to the rate of insect damage that ranged from 36.67% to 13.33%, respectively. Agronomic traits of six transgenic sugarcane lines with medium copy numbers were similar to the non-transgenic parental line. However, phenotype was poor in lines with high or low copy numbers. Compared to the non-transgenic control plants, all transgenic lines with medium copy numbers had relatively equal or lower sucrose yield and significantly improved sugarcane borer resistance, which lowered susceptibility to damage by insects. This suggests that the transgenic sugarcane lines harboring medium copy numbers of the *cry1Ac* gene may have significantly higher resistance to sugarcane borer but the sugarcane yield in these lines is similar to the non-transgenic control thus making them superior to the control lines.

## Introduction

Sugarcane (*Saccharum officinarum* L.) is an important sugar crop that is widely cultivated in the tropical and subtropical regions. It provides about 80% of the world sugar [1] and more than 92% of sugar in China [2]. In addition, sugarcane is also a major raw material for ethanol production in countries such as USA and Brazil, and accounts for nearly 90% of the feedstock used in ethanol production [3]. Equally important is the sugarcane borer, *Diatraea saccharalis* (F.), which is one of the most important lepidopteran pests attacking sugarcane plants and causing more than 10% loss in sugarcane yield worldwide [4]. Damage occurs during the entire crop season and in different tissues resulting in a decreased emergence rate, increased dead heart rate of seedlings, increased stem wind-breakage rate in the adult-plant stage and a reduced sucrose level in the harvest stage. Increasing plant resistance to this insect pest is an effective method to reduce damage by the sugarcane borer. This strategy is also economical and has minimal environmental impact [5,6]. Sugarcane cultivars are complex polyploids with more than 120 chromosomes but without effective insect resistance genes in the sugarcane gene pool [7,8]. This presents a challenge in creating insect-resistant sugarcane cultivars by conventional cross-breeding.

*Cry1Ac* gene, one of the *cry1* genes isolated from *Bacillus thuringiensis* (Bt), codes for an insecticidal crystal protein, which kills lepidopterans upon entry into the alimentary tract [9]. The first successful insect-resistant transgenic tobacco contained the *cry1A(b)* gene introduced through the *Agrobacterium*-mediated transformation [10]. These transgenic tobacco plants were resistant to *Manduca sexta*. Since then, a number of transgenic plants resistant to lepidopterans have been created including tomato [11], cotton [12], potato [13], corn [14], and rice [15]. Transgenic sugarcane was created by firstly introducing the *cry1Ab* gene [7] followed by introducing multiple insecticidal genes such as *cry1A(b)* [16,17], *Galanthus nivalis* agglutinin (GNA) [18,19], soybean proteinase inhibitors [20], *cry1Aa3* [21] and *cry1Ac* [22,23]. Transgenic sugarcane, resistant to the sugarcane borer, was also generated by transferring the *cry1Ab* gene driven by the *CaMV35S* promoter into sugarcane [7]. These transgenic plants resisted insect damage although *cry1A(b)* expression was low. Sugarcane cultivars ROC16 and YT79-177 were created by particle bombardment of a modified *cry1Ac* gene, and about 62% of the transgenic plants were resistant to damage by the stem borer in both greenhouse and field trials [23].

In this study, our goal was to improve sugarcane borer resistance in FN15, a newly released sugarcane cultivar with high sucrose content. We investigated the correlation between resistance to sugarcane borers and the copy number of the *cry1Ac* gene. We determined the level of Cry1Ac protein and investigated how it affected the yield traits and sucrose content. To achieve these goals, the plant expression vector, pGcry1Ac0229 was constructed and introduced into sugarcane by particle bombardment followed by screening and analysis of the transgenic sugarcane lines. It is anticipated that the findings of this study will allow breeding of sugarcanes that are resistant to stem borers.

## Materials and Methods

### Materials

The cassette containing the *cry1Ac* gene, *35s* promoter and *nos* terminator in the cry1AcPRD vector was provided by Prof. Illimar Altosaar, University of Ottawa, Canada. The plant expression vector, pGreenⅡ0229, was obtained from the John Innes Centre in England. The sugarcane cultivar, FN15 used for transformation was provided by the Key Laboratory of Sugarcane Biology and Genetic Breeding, Ministry of Agriculture, Fujian Agriculture and Forestry University, China. All chemicals used were analytical grade.

### Vector construction

The cassette containing the *35s* promoter, *cry1Ac* gene and *nos* terminator was digested from the cry1AcPRD vector using restriction enzymes, *Eco*R I and *Hin*d III. The expression vector, pGreenⅡ0229 was also digested with *Eco*R I and *Hin*d III and linked to the cassette with T_4_-DNA ligase to create a new plant expression vector termed as pGcry1Ac0229 containing the target gene, *cry1Ac* (S1 Fig).

### Transformation and screening

Transformation was performed based on the PDS 1000/He particle gun operating manual (Bio-Rad, Calif., USA). The embryonic calli of the sugarcane cultivar FN15 were derived from transverse segments of young leaf roll region with the apical meristem. They were cut into 1–2 mm thick discs and cultured in MS medium [24] containing 3.0 mg/L dichlorophenoxyacetic acid (2,4-D) in dark for 2–4 weeks, and used for transformation via bombardment. For each bombardment, 15–20 pieces of embryonic calli with a diameter of 2–3 mm were placed in the centre of a culture dish with MS-based induction medium containing 0.2 mol/L sorbitol and 0.2 mol/L mannitol. Then, they were cultured in dark at 28°C for 4–8 h prior to bombardment. Several culture plates were used to bombard the *cry1Ac* genes while only two plates were used as controls. One plate served as the control for bombardment with tungsten particles without plasmid DNA, and the other plate was the no bombardment control. The plasmid, pGcry1Ac0229 was coated on tungsten particles (Bio-Rad, 0.7) before bombardment and approximately 1.0 μg of DNA was used for each bombardment. After the procedure, the calli were spread over the surface of the medium and cultured for 1 d at 28°C in dark. Then, the calli were transferred to the MS induction medium containing 2.0 mg/L 2,4-D and 0.75 mg/L phosphinothricin (PPT) and cultured in dark for 2 weeks. For regeneration culture, the calli were transferred to a MS regeneration medium containing 1.0 mg/L 6-Benzyladenine (6-BA), 1.0 mg/L Kinetin (KT) and 0.75 mg/L PPT, and cultured for several cycles of alternating light and dark conditions for 2 weeks at 28°C until sugarcane plantlets regenerated. The plantlets were then transferred to a ½MS rooting medium containing 3.0 mg/L 1-naphthlcetic acid (NAA) until roots developed. Viable putative transformants were transferred to soil in the greenhouse. When the putative transformants grew 5–8 cm in height, they were sprayed with a 3‰ (V/V) Basta (active ingredient phosphinothricin /glufosinate ammonium) solution for preliminary screening.

### DNA extraction and primer design

Total genomic DNA was extracted from fresh young leaves of the Basta-resistant plantlets using a CTAB method described previously [25]. The negative control was the FN15 non-transgenic sugarcane without bombardment. All DNA samples were stored at -20°C after measuring the DNA concentration by a UV absorption method. DNA purity was analyzed by the A_260_/A_280_ ratio, while the integrity was evaluated by electrophoresis. Primer pairs for polymerase chain reaction (PCR) and real-time quantitative PCR (RT-qPCR) were designed by Primer Premier 5 software. Based on the sequence of the *cry1Ac* gene, a pair of primers was designed for the amplification of a 211 bp fragment (forward: TATCGTTGCTCAACTAGGTCAGGGTGTC, and reverse: CATTGTTGTTCTGTGGTGGGATTTCGTC) for PCR, and another pair to amplify a 74 bp fragment by RT-qPCR as follows: 1ACQF: 5’-ACCGGTTACACTCCCATCGA-3’, 1ACQR: 5’-CCAGCACCTGGCACGAA-3’, 1ACProbe: 5’- (FAM) TCTCCTTGTCCTTGACACAGTTTCTGCTCA-3’(TAMRA).

### PCR analysis

PCR was performed using Eppendorf 5331 (Eppendorf Company, Hamburg, Germany). Each reaction volume was 25 μL containing 2.5 μL PCR buffer (10×), 2.0 μL dNTP mix, 1.0 μL (100 ng μL/L) template DNA, 1.0 μL (10 μmol/L) each of the forward and reverse primers, 0.125 U of Taq DNA polymerase (Takara, Dalian, China), and ddH_2_O. Following the initial polymerase activation at 95°C for 5 min, 30 cycles were performed at 95°C for 30 s, 56°C for 30 s and 72°C for 30 s with a final extension at 72°C for 10 min. The amplified products were detected on a 1.5% (w/v) agarose gel.

### Copy number calculation in transgenic sugarcane lines by RT-qPCR analysis

RT-qPCR was performed using an ABI PRISM 7500 Sequence Detection System (Foster, USA). The reaction volume was 25 μL and contained 12.5 μL of Fast Start Universal Probe Master Mix, 1.0 μL of diluted genomic DNA (25 ng), 1.0 μL (10 μmol/L) each of the forward and reverse primers and sterile ddH_2_O. The following reaction conditions were used; 50°C for 2 min, 95°C for 10 min, 40 cycles of 95°C for 15 s, 60°C for 1 min; 1 cycle of 95°C for 15 s, 60°C for 15 s, 95°Cfor 15 s. Each sample had three replications. In parallel, the pGcry1Ac0229 plasmid DNA was serially diluted to 10^8^, 10^7^, 10^6^, 10^5^, 10^4^, 10^3^, 10^2^, and 10^1^ copies/μL and subjected to RT-qPCR with each reaction having three replications. The plasmid copy number was obtained using the following formula: plasmid copy number (copies/μL) = 6.02 × 10^23^ (copies/mol) × plasmid concentration (g/μL)/ plasmid molecular weight (g/mol)/660 [26]. Each copy number corresponding to the C_t_ value was obtained after the reaction. Then, using the lg copies in the X axis, and the C_t_ value in the Y axis, the standard curve was established to obtain the linear equation Y = kX+b. The total copy number (10^Xt^) was calculated by relating the C_t_ value (Y_t_) and linear equation for the *cry1Ac* transgenic lines in each reaction. Then, the single cell copy number of each sample was calculated using the following formula: Copies/genome = 10^Xt^/[25 n g×10^−9^×6.02×10^23^/ (10,000 (M bp)×10^6^×660)] [27].

### Southern blot analysis

The PCR-positive transgenic sugarcane lines were analyzed by Southern blotting. Primers (forward: TATCGTTGCTCAACTAGGTCAGGGTGTC and reverse: CATTGTTGTTCTGTGGTGGGATTTCGTC) were used to amplify the *cry1Ac* fragment to prepare the labeled probe using the PCR DIG Probe Synthesis Kit (Roche, Switzerland). About 40 μg of genomic DNA was extracted from fresh young leaves of every transgenic sugarcane line and non-transgenic control, and was completely digested with the restriction enzyme *Hin*d III, which is present once in pGcry1Ac0229. Southern blotting was performed based on the DIG DNA Labeling and Detection Kit manual.

### ELISA analysis

ELISA was used to detect Cry1Ac protein expression in transgenic sugarcane lines according to the manufacturer’s instructions in the Qualiplat Kit. Total protein from fresh mature sugarcane leaves and stems was used in ELISA. The cry1Ac crystal protein standard was purchased from Envirologix (Portland, USA) and serially diluted to 5.0 ppb, 2.5 ppb, 1.25 ppb, 0.625 ppb, 0.3125 ppb and 0.15625 ppb with 1×phosphate buffer saline (pH 7.4). The non-transgenic sugarcane line without particle bombardment was used as the negative control. Sterile ddH_2_O served as the blank control. Each sample had three replications. OD_450_ value of the standard was obtained after the reaction followed by plotting the concentration of standard in the X axis and the OD_450_ value in the Y axis to establish the standard curve and form the linear equation y = kx + b. Cry1Ac protein expression (X_t_) in the transgenic sugarcane lines was calculated by relating the OD_450_ value (Y_t_) to the standard curve.

### Trial design and investigation of phenotype traits in sugarcane lines

In isolated field trials, 14 different transgenic *cry1Ac* sugarcane lines and the non-transgenic controls were evaluated during 2012 to 2013 using a randomized complete block design with three replications. Each block had three 8 m-long rows with 1.3 m space between rows, covering an area of 31.2 m^2^. The sugarcane crops were harvested in January the following year. Field management was slightly better than commercial cultivation. The fertilizers applied were: nitrogen (N) at 345 kg/ha, phosphorus (P_2_O_5_) at 240 kg/ha and potassium (K_2_O) at 360 kg/ha. At mature stage, yield estimates were obtained from the stalks in the middle rows. The estimates included height, stem diameter, and brix in ten consecutive principal stalks from 10 plants. The number of millable stalks in 5 m long rows was also counted in each block. The cane yield per unit area was calculated based on the area, and number and weight of stalks. Sucrose yield was calculated based on the average sucrose content and cane yield using equations (1): Sucrose content (%) = brix × 1.0825–7.703; and (2): Sucrose yield (t/ha) = Cane yield (t/ha) × Sucrose content (%)

### Resistance of transgenic sugarcane to sugarcane borer

Stem segments from transgenic and non-transgenic lines were pre-germinated in an incubator. Upon reaching the 4–5 leaf stage, two 8 d old larvae were placed on each seedling. A total of 8–10 seedlings were infested for each line and maintained in isolation using fly netting. The assay was repeated 3 times. In the field trials, the percentage of stalks damaged by the sugarcane borer and damage symptoms in the attacked leaves and internodes were recorded along with agronomic traits.

## Results

### Construction of pGcry1Ac0229

Successful construction of the recombinant plasmid, pGcry1Ac0229, was first verified by a single digestion with *Hin*d III and a double digestion with *Hin*d III and *Eco*R I, which yielded the expected 7,238 bp, and 4,436 bp and 2,802 bp bands, respectively (S2 Fig). Further sequencing demonstrated that this recombinant plasmid had the expected expression cassette inframe (results not shown).

### Particle bombardment and resistance screening

**Fig 1:**
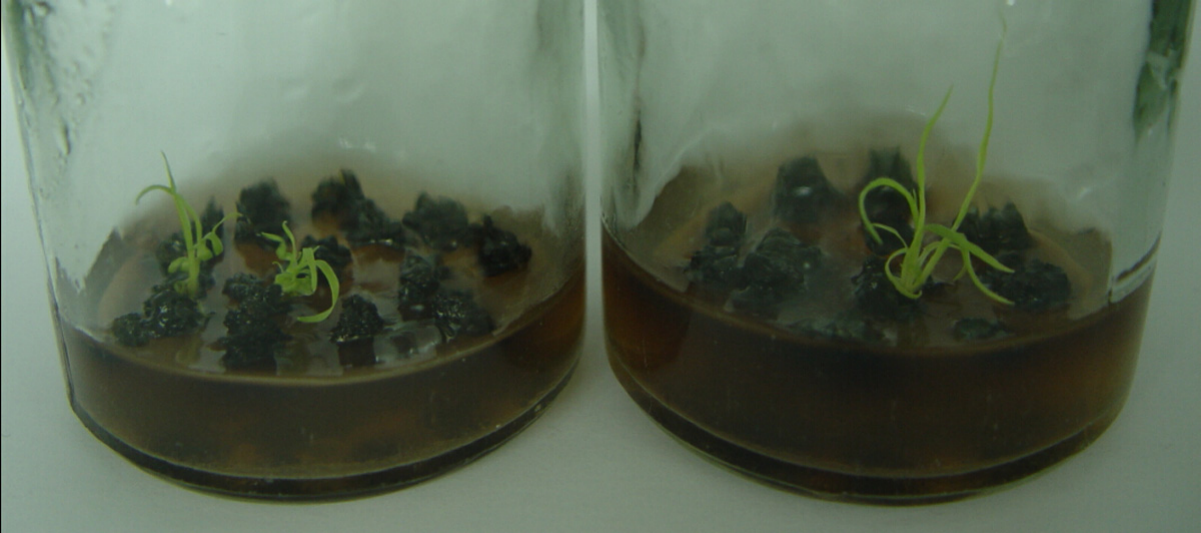
Partially regenerated plantlets with PPT resistance in differentiation culture.

The pGcry1Ac0229 plasmid DNA was used to prepare microprojectiles for bombardment transformation, and the embryonic calli of the sugarcane cultivar FN15 were used as receptors. Based on previously determined screening concentrations of PPT (0.75 mg/L), cultures were screened and the putative transformants that survived the stress of PPT were obtained. Wild type cells without PPT resistance gradually turned brown and then died (Fig 1). Regenerated plantlets were transferred to rooting medium without PPT and the total number of survivors was 189. These plantlets were planted in soil in 72-cave trays and the second round of screening *in vitro* was conducted by spraying a solution containing 3.0 ‰ Basta on the plantlets. After 15–20 d, most of them gradually became yellow, wilted, and dried (S3 Fig). The final total of surviving plants was 26.

### PCR identification of resistant sugarcane plants

**Fig 2:**
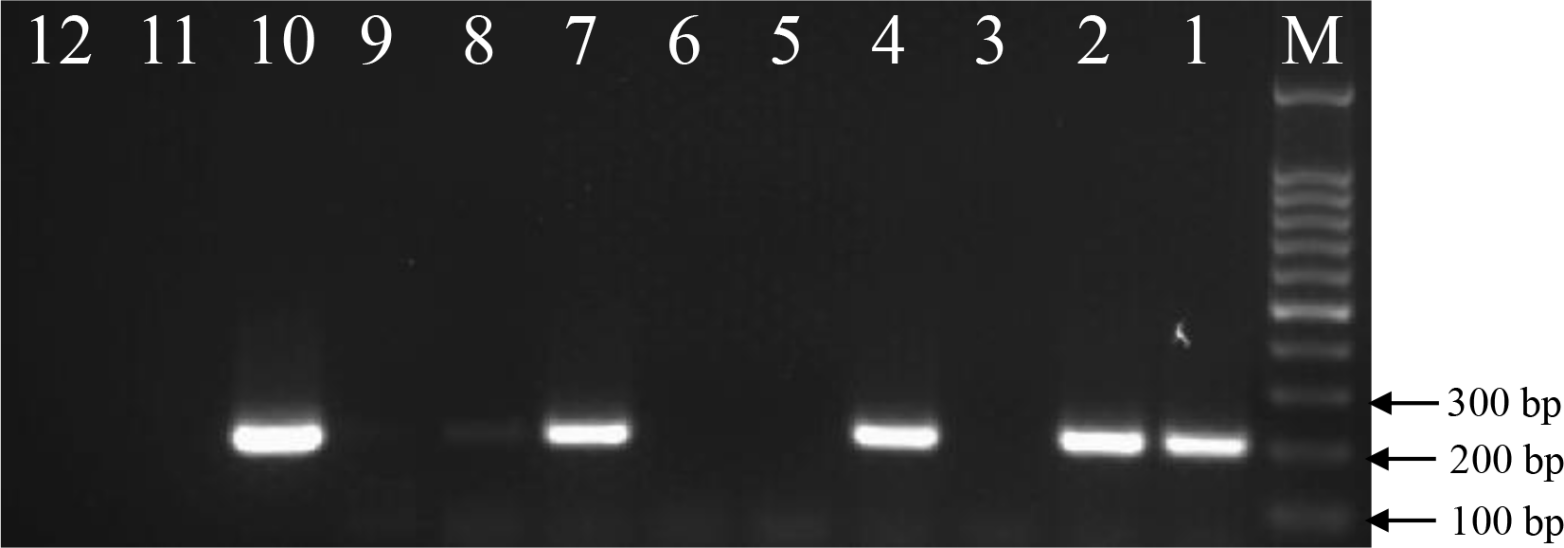
Electrophoresis of PCR amplified products of the candidate *cry1Ac* transgenic sugarcane plants. M, DNA Marker; 1–9, Basta-resistant plants; 10, Positive control (plasmid pGcry1Ac0229); 11, Negative control (non-transgenic sugarcane without bombardment); 12, Blank control.

PCR validation was performed on the Basta resistant regenerated plants. A total of 14 independent plants amplified the expected size DNA consistent with the positive control (pGcry1Ac0229), while the negative non-transgenic line and the blank control did not amplify any band (Fig 2). Further sequencing showed that this band was precisely the 211 bp partial sequence of *cry1Ac*, suggesting that the *cry1Ac* gene was successfully inserted into the sugarcane genome. A total of 14 among the 26 Basta-resistant regenerated plants tested positive by PCR.

### Copy number calculation in transgenic sugarcane plants by RT-qPCR

Fourteen Basta-resistant and *cry1Ac* gene positive transgenic sugarcane plants were further tested using RT-qPCR to determine *cry1Ac* gene copy number. A standard curve for quantitative detection of *cry1Ac* gene based on RT-qPCR was constructed (S4 Fig). The slope was Y = -3.140X+43.622, R^2^ = 0.998, where Y represents C_t_ value and X represents the log of starting template copy number. The C_t_ (18~40) value correlated well with the copy number of the starting template (10^1^~10^8^) (R^2^ = 0.998). According to the linear equation above, the total copy number (10^Xt^) of the target gene in samples was calculated by relating the C_t_ value (Y_t_). The copy number of *cry1Ac* gene inserted into a single cell was calculated according to the formula (Table 1):

$$\text{Copies}/\text{genome}=\;10^{\text{X}\;\text{t}}/\left[25\;\text{ng}\times 10^{-9}\times 6.02\times 10^{23}/\;\left(10,000\times 10^{6}\times 660\right)\right]$$

**Table 1:** Copy number estimation in different transgenic sugarcane lines with a *cry1Ac* gene.

Sugarcane lines	C _t_ Ⅰ	C _t_ Ⅱ	C _t_ Ⅲ	C _t_ mean	Copy number
T-1	30.82	30.95	30.76	30.85±0.10	5.13
T-2	27.75	27.61	27.87	27.74±0.13	49.92
T-3	27.35	27.35	27.64	27.45±0.17	62.08
T-4	32.10	32.15	31.91	32.06±0.13	2.11
T-5	27.81	27.83	28.03	27.89±0.12	44.78
T-6	33.12	32.73	33.02	32.96±0.20	1.09
T-7	31.74	32.10	32.06	31.96±0.20	2.26
T-8	28.82	28.82	28.70	28.78±0.07	23.29
T-9	26.62	26.51	26.66	26.60±0.08	115.57
T-10	26.93	27.06	27.12	27.04±0.10	83.68
T-11	28.80	29.13	29.52	29.15±0.36	17.78
T-12	26.72	26.66	26.83	26.73±0.09	104.56
T-13	26.12	26.37	26.29	26.26±0.13	147.92
T-14	27.29	27.38	27.68	27.45±0.21	61.86
CK FN15	34.84	34.65	34.89	34.79±0.13	0.28

The C_t_ values of the three replicates had small standard deviations in the different transgenic lines (Table 1). The copy number in the 14 transgenic sugarcane plants ranged from 1 to 148, and they were divided into three groups: high copy number (>80), medium copy number (10~80) and low copy number (<10).

### Copy number of transgenic sugarcane lines detected by Southern blot analysis

Based on the copy number determined by RT-qPCR analysis, six independent transgenic lines, including two high (T-9, T-12), two medium (T-2, T-11) and two low (T-1, T-4) copy number lines, were selected for copy number detection by Southern blot analysis. There was no hybridization signal in the two transgenic lines with low copy number and in the non-transgenic control; 3–4 copies were observed in the two medium copy number lines; and 5–6 copies were observed in the two high copy number lines.

### ELISA analysis of Cry1Ac protein content in transgenic sugarcane lines

**Fig 3:**
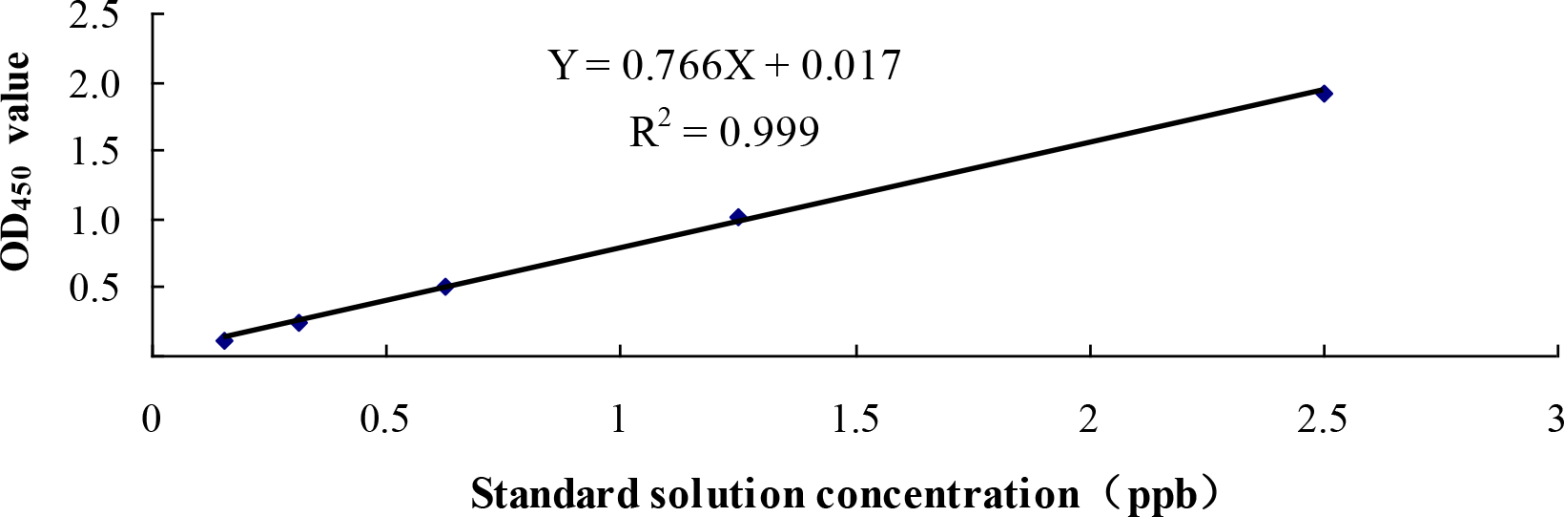
The standard curve of Cry1Ac protein constructed by ELISA.

**Fig 4:**
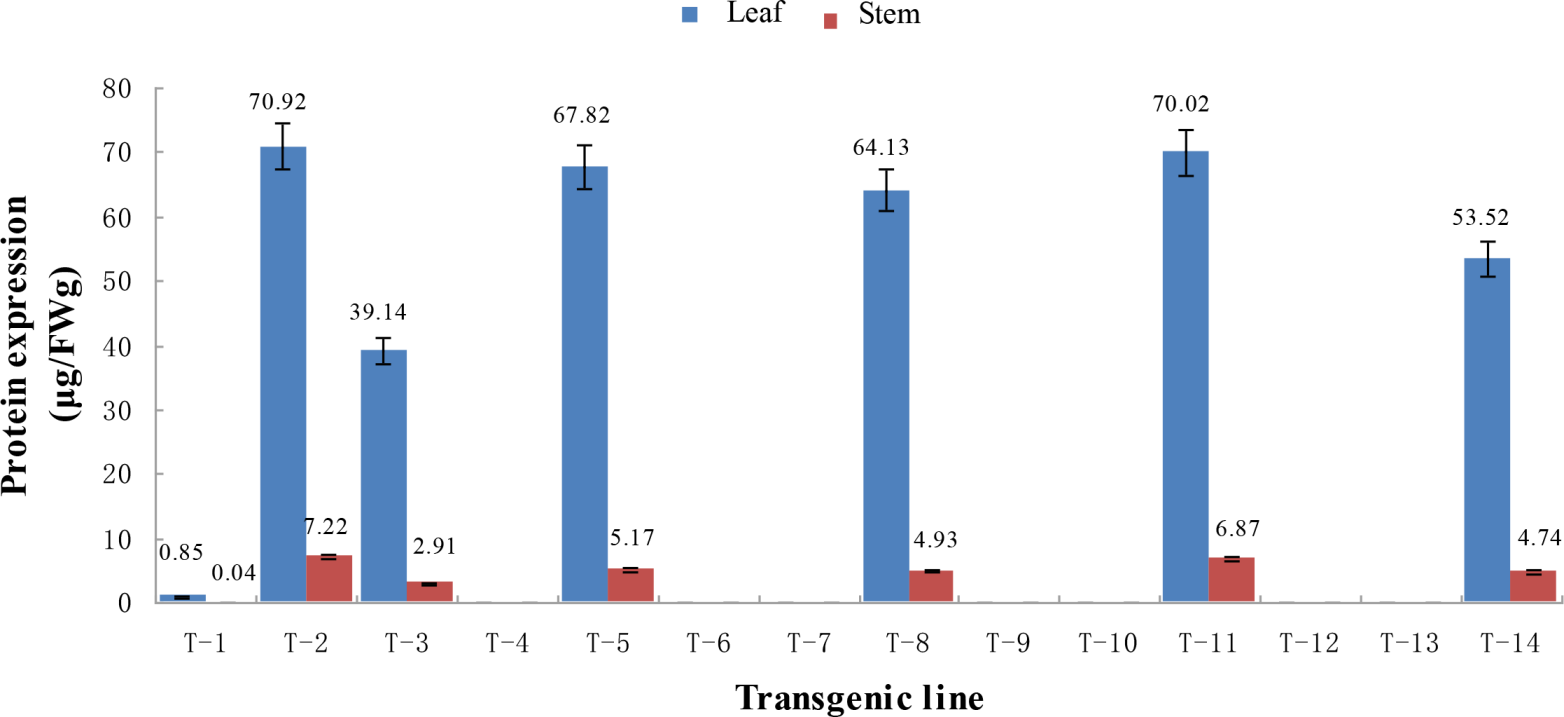
Cry1Ac protein expression in the leaves and stems of 14 different transgenic sugarcane lines detected by ELISA.

Double-antibody sandwich ELISA was performed to determine the Cry1Ac protein content in mature stage leaves and stems of 14 transgenic sugarcane lines. Standard *Bt* samples were used to construct a standard curve for quantitative protein detection (Fig 3). The slope was Y = 0.766X+0.017, R^2^ = 0.999, where Y is absorbance at OD_450,_ and X is the concentration of standard *Bt* Cry1Ac protein samples. Significant correlation was found between the absorbance and the concentration of the standard protein (R^2^ = 0.999). Thus, the protein expression of the tested samples was measured based on the linear equation (Fig 4). The results showed that Cry1Ac protein expression was only detected in leaves and stems of seven transgenic lines. Expression in leaves was variable and ranged from a minimum of 0.85μg/FWg in T-1 to a maximum of 70.92 μg/FWg in T-2. In stems, the expression was also variable and ranged from a minimum of 0.04 μg/FWg in T-1 to a maximum of 7.22 μg/FWg in T-2. It should be noted that although there was no Cry1Ac protein expression in the remaining seven transgenic lines, the expression of *cry1Ac* gene was detected in all these lines.

### Agronomic and industrial traits in transgenic sugarcane lines

Agronomic and industrial traits of 14 transgenic lines and the control sugarcane cultivar FN15 were investigated in mature stage plants. As shown in Table 2, univariate statistical analysis indicated that stalk height in transgenic lines T-2, T-3, T-5, T-8, T-11, and T-14 was similar to the control line and no significant difference was observed. Although in some cases slightly higher or lower heights were observed, eight lines had significantly lower heights than the control. Stalk diameter of transgenic lines T-2 and T-3 was not significantly different from the control while in the remaining 12 lines it was significantly less than the control. For brix, only line T-4 was not significantly different compared to the control but the remaining 13 lines were significantly different. Lines T-1, T-6, T-7, T-9, T-10, T-12, and T-13 had significantly higher brix and lines T-2, T-3, T-5, T-8, T-11, and T-14 significantly lower brix than the control. With respect to millable stalks in a block, T-2, T-3, T-5, T-8, T-11, and T-14 had higher and T-1 had lower numbers than the control although there was no statistical significance among these values when compared to the control. The theoretical sucrose yield was calculated based on the height, stalk diameter, brix, and millable stalks in a block. These values were marginally lower in lines T-3, T-8, and T-11 but not significantly different from the control, while the remaining lines had significantly lower values than the control.

**Table 2:** Agronomic characteristics, industrial traits and the stalks borer damage percentage in transgenic sugarcane lines and the non-transgenic control.

Transgenic line	H(cm)	D (cm)	Brix (%)	SNB	TSY (kg/block)	TSY (t/ha)	SDR (%)
**T-1**	170.67±5.21 b	2.50±0.07 ef	22.73±0.06 ab	184.62±4.62 bc	26.21±2.91 d	8.40±0.93 d	36.67±5.77 bcd
**T-2**	207.42±2.16 a	3.05±0.06 abc	19.43±0.15 f	227.69±2.66 a	46.1±3.45 bc	14.78±1.11 bc	13.33±5.77 e
**T-3**	213.50±3.88 a	3.20±0.12 ab	19.61±0.30 ef	221.54±7.99 a	51.65±5.19 ab	16.56±1.66 ab	21.67±2.89 cde
**T-4**	172.89±0.51 b	2.41±0.06 f	21.11±0.14 c	152.31±9.23 de	18.1±0.54 de	5.80±0.17 de	43.33±5.77 bc
**T-5**	207.92±4.42 a	2.87±0.07 cd	19.49±0.22 ef	224.62±7.05 a	40.52±3.41 c	12.99±1.09 c	15.00±5.00 de
**T-6**	181.11±2.01 b	2.37±0.03 f	22.34±0.16 ab	166.15±4.62 cd	21.82±0.37 de	7.00±0.12 de	46.67±15.28 b
**T-7**	172.89±1.26 b	2.56±0.04 ef	22.82±0.25 a	156.92±9.23 de	23.74±2.16 de	7.61±0.69 de	56.67±5.77 ab
**T-8**	203.92±1.66 a	3.16±0.04 ab	19.92±0.24 def	226.15±4.62 a	50.11±2.63 ab	16.06±0.84 ab	16.67±5.77 de
**T-9**	150.00±2.19 c	2.65±0.04 e	22.33±0.21 ab	133.85±12.21 ef	18.2±1.72 de	5.83±0.55 de	43.33±5.77 bc
**T-10**	135.67±4.58 d	2.50±0.01 ef	22.70±0.18 ab	141.54±9.61 ef	15.88±1.21 e	5.09±0.39 e	53.33±11.55 ab
**T-11**	203.50±4.27 a	3.00±0.10 bc	20.24±0.10 d	229.23±7.05 a	46.68±2.2 abc	14.96±0.71 abc	14.33±1.15 e
**T-12**	145.00±1.20 cd	2.66±0.05 de	22.37±0.23 ab	153.85±9.61 de	20.42±1.35 de	6.54±0.43 de	53.33±5.77 ab
**T-13**	143.67±3.48 cd	2.54±0.06 ef	22.19±0.10 b	124.62±9.23 f	14.86±1.45 e	4.76±0.46 e	46.67±11.55 b
**T-14**	211.25±10.13 a	2.92±0.08 c	20.09±0.29 de	220.00±2.66 a	43.67±1.7 bc	14.00±0.55 bc	18.33±5.77 de
**CK FN15**	209.92±4.14 a	3.25±0.14 a	21.47±0.25 c	206.15±9.61 ab	55.86±7.64 a	17.91±2.45 a	72.67±2.52 a

Notes: (1) H: stalk height; D: stalk diameter; SNB: stalk number per block; TSY: theoretical sucrose yield; SDP: stalks borer damage percentage. (2) Data followed by different letters indicate significant difference at 0.05 level (Duncan Test); (3) The area of sub domain is 31.2 m^2^

### Transgenic sugarcane lines are resistant to sugarcane borer

**Fig 5:**
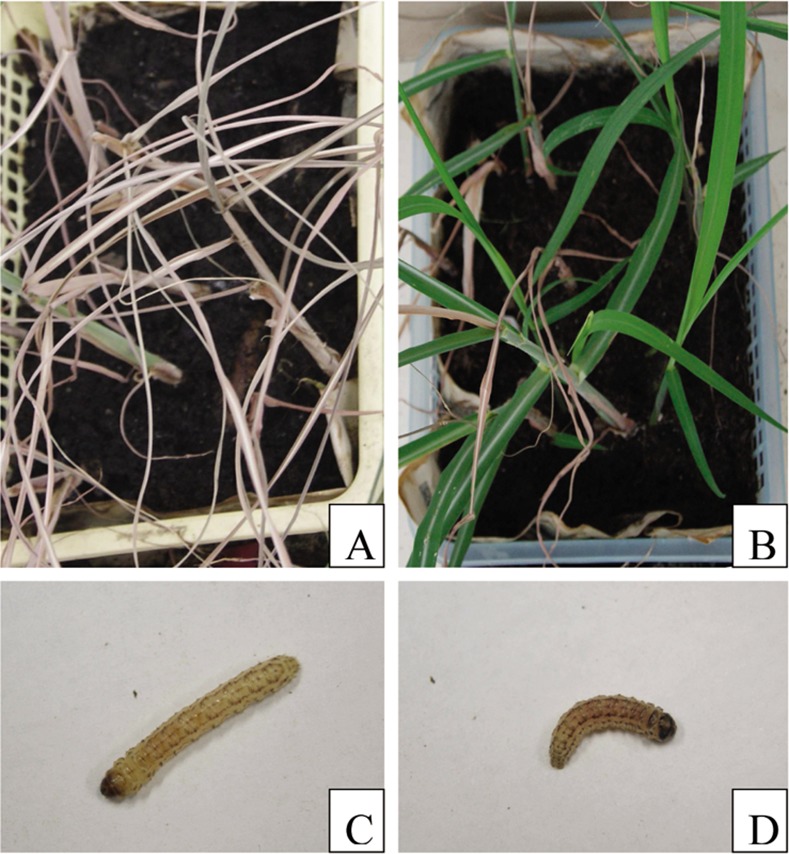
Bioassay in the seedling stage. **A**: Symptoms of non-transgenic sugarcane plantlets challenged with sugarcane borers; **B**: Symptoms of transgenic sugarcane plantlets challenged with sugarcane borers; **C**: Sugarcane borer feeding with non-transgenic sugarcane plantlets; **D**: Sugarcane borer feeding with transgenic sugarcane plantlets.

**Fig 6:**
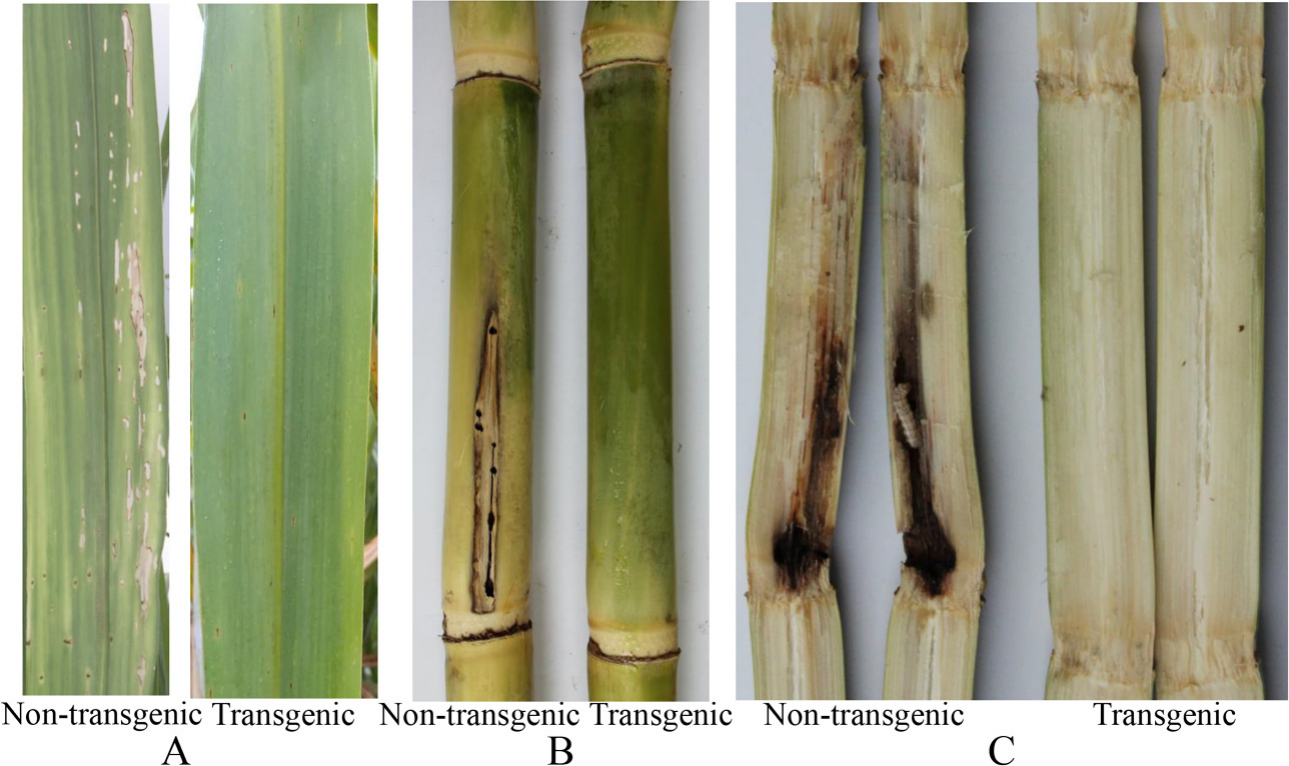
Typical symptoms caused by natural stem borer infestation in the field. **A**: Leaf symptoms; **B**: Stem symptoms (aspect); **C**: Stem symptoms (inside stalk).

**Fig 7:**
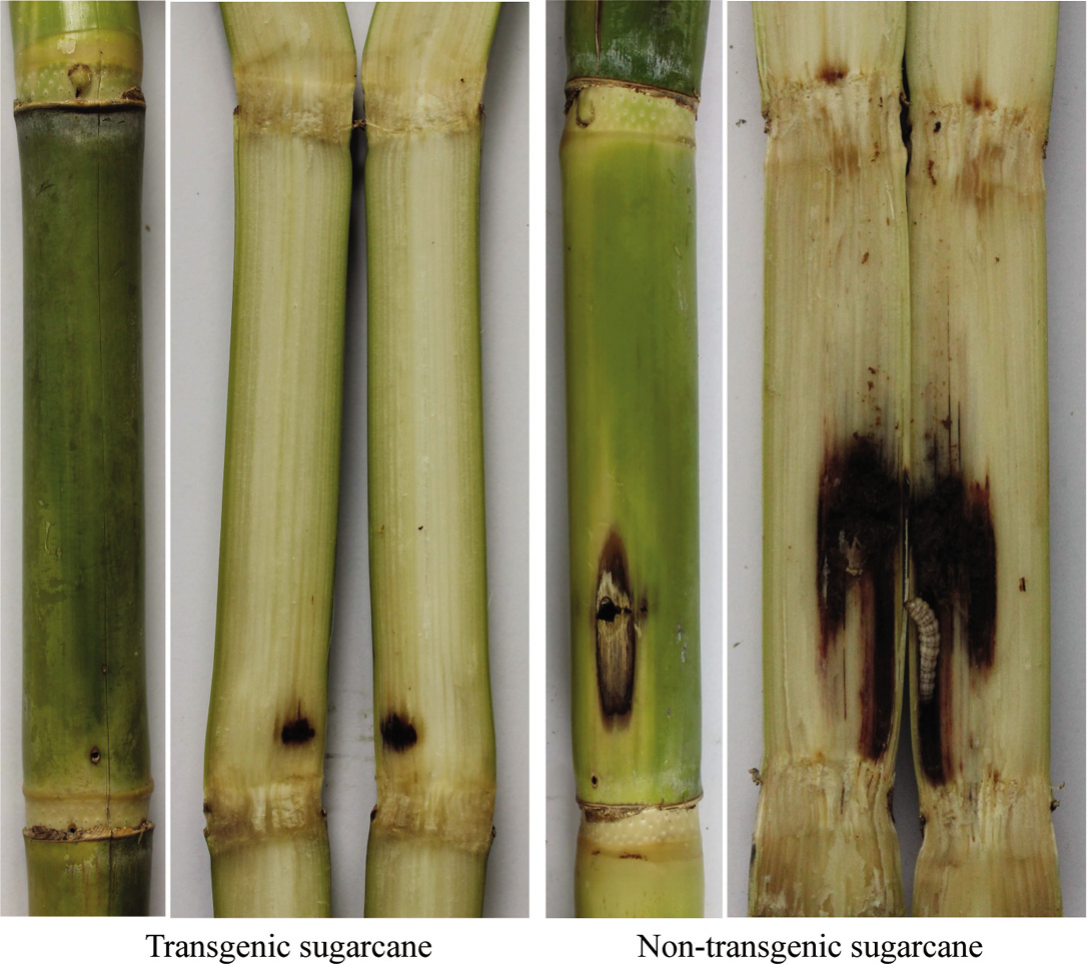
Damage comparison between transgenic and non-transgenic sugarcane stalks.

The transgenic sugarcane lines were generally more resistant to sugarcane borer compared to the non-transgenic lines. In the seedling stage, 7 d after inoculation, the typical effects of borer attack leading to dead heart were seen on the non-transgenic plants. The same effects were observed after 10 d in several transgenic lines. Plantlets of non-transgenic sugarcane withered slowly 10 d after inoculation. At 15 d, only transgenic lines continued to grow normally (Fig 5B), while non-transgenics withered or died (Fig 5A). In the transgenic lines, only 10%-20% of borers survived but these appeared weak and were small (Fig 5D). In contrast, the borers in non-transgenic control plants had significantly higher survivorship and were larger (Fig 5C). In the field trials, the transgenic lines were also resistant to the sugarcane borer damage to leaves and stems compared to the non-transgenic control (Fig 6). The borer damage rate in all transgenic lines was lower than the control and differences were significant except for lines T-7, T-10, and T-12. There was no significant difference between the lines T-2, T-3, T-5, T-8, T-11, and T-14, all of which had higher protein expression and a medium *cry1Ac* copy number (Table 2). Although the transgenic sugarcane lines were still attacked by stem borers, the degree of damage was clearly less than the damage done to the non-transgenic control line (Fig 7).

## Discussion

We constructed the plant expression vector pGcry1Ac0229 containing two expression cassettes, *d35s*-*AMV*-*cry1Ac*-*nos*, and *Nos-bar-nos*. The target gene *cry1Ac* was placed under the control of the constitutively expressed double promoter *35s* and the enhancer *AMV*. These two regulatory sequences significantly increase the expression of exogenous genes in transgenic plants [28]. Consistent with this, the Cry1Ac protein content in the leaves of transgenic sugarcane lines ranged from 0 μg/FWg to 70.92 μg/FWg. A previous study reported that the expression of *cry1A(b)* regulated by *CaMV35S* yielded only a maximum of 27.23 ng/mg (i.e. 27.23 μg/FWg) cry1A(b) protein in transgenic sugarcane leaves [7]. This suggests that the double promoter *35s* and the enhancer *AMV* can significantly enhance the expression of target genes.

The *Bar* gene is a high efficiency selectable marker in plant genetic transformation. It has been widely used in many plant species [29,30] including sugarcane [17,31,32] due to its advantage in screening putative transformants. Sugarcane is more sensitive to PPT than G418 and hygromycin (Hyg) [33]. The screening concentrations of PPT for the sugarcane cultivar FN81-745 and Badila in tissue culture were 0.75–1.0 mg/L PPT, 30 mg/L G418 and 30 mg/L hygromycin [33]. The screening concentration of PPT in the callus differentiation stage of the sugarcane cultivar FN95-1702 (i.e. FN15) was 0.5 mg/L [34]. Putative transformants surviving in tissue culture could be re-screened by spray treatment with Basta herbicide at the seedling stage after soil transplantation. This procedure allows rapid and cost effective identification of transformants because the leaves of transgenic plants will remain green but leaves from non-transgenic leaves turn yellow, wither, and die. In this study, we used 0.75 mg/L PPT to screen the sugarcane cultivar FN15 in callus subcultures and differentiation culture stages. Screening was not performed at the rooting stage. This protocol allowed rapid and normal rooting of putative transformants. It also provided a relatively relaxed selection procedure due to fast growth thus shortening the screening time by 4–6 weeks at the tissue culture stage. A more stringent selection was performed in plantlets on seedling growth stage using 3‰ (V/V) Basta, which resulted in efficient identification of *cry1Ac* transgenics. Additionally, the *bar* gene, coding the bialaphos/phosphinothricin resistance protein, is resistant to PPT, the active ingredient in Basta or Glufosinate and Bialaphos in Herbiace [35]. Transgenic plants containing the *bar* gene are resistant to Basta or Glufosinate and Herbiace. This is also an important agronomic character for sugarcane with respect to weed management due to the slow early stage growth of sugarcane. Thus, transgenic sugarcanes with the *bar* gene have great potential for commercial use.

It is important to determine the copy number of transgenes in transgenic lines because copy number can affect genetic stability and expression level. The traditional method to estimate copy number of exogenous genes in transgenic plants is by Southern blot analysis. Recently, RT-qPCR technology has been widely used to determine the copy number of exogenous genes [36,37] but the results can be inconsistent with Southern blot analysis. Using quantitative fluorescence PCR, Weng *et al*. [38] observed that the copy number of *gus* and *npt II* genes in 7 out of 10 transgenic events in *Brassica napus* was consistent with Southern blot analysis. Cheng *et al*. [39] tested the exogenous gene, *gfp* in tetraploid upland cotton transgenic lines and showed that the copy number detected by quantitative fluorescence PCR was greater than that observed by Southern blot analysis. Yang *et al*. [40] analyzed the copy number of *npt II* in transgenic cotton and found that the copy number detected by Southern blot analysis was less than or equal to that estimated by qPCR analysis. In this study, we established a standard curve to estimate the copy number of *cry1Ac* gene by SYBR Green quantitative fluorescence PCR. The slope of the reaction was -3.140 and correlation coefficient was 0.998 suggesting a strong correlation between the Ct value and the copy number of the starting template. We then determined the copy number in transgenic lines and found that they ranged from 1 to 148. Because the chromosome ploidy of modern sugarcane is complex and remains undefined [41], the actual ploidy in modern sugarcane varieties including the test cultivar FN15 used here is unclear. Transgenic sugarcane lines were obtained by particle bombardment, which normally introduces multiple copies of exogenous gene expression cassettes and integrates them into the transgenic plant genome [42]. To verify the copy number of the transgenic sugarcane lines, six different transgenic lines with high (>80), medium (10~80) or low (<10) copy numbers of the *cry1Ac* gene were selected for Southern blotting. The copy numbers estimated by RT-qPCR were higher than those estimated by Southern blotting, which was consistent with previous studies on transgenic rice [43] and cotton [39]. However, the trend in copy number estimation by the two methods was consistent. Inconsistency in copy number estimation by the two methods may result from the following two factors. First, multi-copy tandem integrations in transgenic lines, where the estimated copy number is based on southern blot analysis, are typically lower than the actual number [40,43]. Second, southern blots may lead to poor resolution of the blotting bands and allow detection of only a few of the total bands. When RT-qPCR is used to estimate exogenous gene copy number, the results can be influenced by the slope and correlation coefficient of the standard curve, as well as the concentration of the starting template. In this study, we established a good standard curve with a slope of -3.140 and a correlation coefficient of 0.998 that supports the accurate estimation of the *cry1Ac* gene copy number.

Protein expression level of exogenous genes can directly affect the target trait and its practical value [17,23]. Transgenic sugarcane lines containing the truncated insecticidal gene *m-cry1Ac* or the partially modified *s-cry1Ac*, were previously evaluated to determine integration sites, transgene expression pattern, and level of resistance to insects [23]. Among the 55 PCR positive plants, 17 were determined to be positive using protein analysis and contained a range of 2.2 ng/mg to 50 ng/mg Cry1Ac protein in leaves and 6 to 8 copies of the *cry1Ac* gene estimated by Southern blot analysis. In our study, there were 14 PCR positive transgenic sugarcane lines but ELISA detected Cry1Ac protein in only 7 lines. Interestingly, RT-qPCR estimation of copy number and ELISA analysis of Cry1Ac protein revealed only one line with low copy number (5 copies) and low Cry1Ac protein content (0.85 μg/FWg in leaf or 0.04 μg/FWg in stem) while the remaining 6 lines had a medium copy number (17~62 copies) and high protein expression (39.14 μg/FWg to 70.92 μg/FWg in leaves or 2.91 μg/FWg to 7.22 μg/FWg in stems). Meanwhile, linear relationship was not observed between the Cry1Ac protein expression level and the copy number of *cry1Ac* gene in the 6 transgenic lines, T-2, T-3, T-5, T-8, T-11, and T-14 with higher protein expression, which is consistent with previous studies [44,45]. This also indicated that expression of the exogenous gene was likely affected by position effects. It should be noted that Cry1Ac protein was not detected in 7 *cry1Ac* transgenic sugarcane lines including 3 lines with low copy number (1–2 copies) and 4 lines with high copy number (83–148 copies). Similarly, protein expression was not detected in lines with low copy number (1–2 copies), which may due to a large sugarcane genome [46]. In contrast, a high copy number of an exogenous gene in transgenic plants could result in co-suppression due to multi-copy integration, thus resulting in transgenic silencing [47,48]. The phenomenon of transgenic silencing has been documented in sugarcane [49,50].

Bt protein expression can improve pest resistance in transgenic plants [17,23,51]. The *cry1Ab* gene has been previously introduced into sugarcane cultivars, Co 86032 and CoJ 64 [17]. Cry1Ab protein in different transgenic events ranged from 0.007% to 1.73% of the total soluble protein in leaf. Transgenic plants had significantly less dead heart at the seedling stage and there was a negative correlation between protein expression and the dead heart rate. Weng *et al*. [23] analyzed pest resistance of *cry1Ac* transgenic sugarcane, and found resistance only in lines expressing the Cry1Ac protein more than 9 ng/mg. A positive correlation between the Cry1Ac content and pest resistance was also observed. Lines with low Cry1Ac protein content (1.8 ng/mg) were more susceptible to pests. In the present study, the percentage of damaged stalks ranged from 13.33% to 36.67% in 7 transgenic sugarcane lines expressing Cry1Ac protein, while in the untransformed control line the damage was as high as 76.67%. These results demonstrated that higher Cry1Ac protein expression results in reduced damage. T-2 line, with the highest protein expression in leaves (70.92 μg/FWg) and stems (7.22 μg/FWg) had the lowest percentage of stalks damaged (13.33%), which was consistent with a previous report [23]. Nevertheless, T-1 line with lower expression of Cry1Ac in leaves (0.85 μg/FWg) and stems (0.04 μg/FWg) was also resistant to borer damage (36.67%) which was less than the control (76.67%) and differed from the results reported by Weng *et al*. [23]. There were limitations in the worm damage evaluation methods used in this study. Assessment was based on the number of wormholes and not the degree of damage. Nevertheless, there was still 13.33% damage in the T-2 line but the damage was confined to the outside of the stalks. In non-transgenic lines, the borers tunneled inside the stalk where they cause relatively more damage (Fig 7).

Weng *et al*. [23] observed that the agronomic traits of stalk growth and juice quantity were severely affected in transgenic lines compared to the control, but the industrial traits of sucrose content, brix, and purity were not significantly different between the control and transgenic lines. In the present study, we found that the transgenic plant lines had variable height, stalk diameter, millable stalks, and brix. Some transgenic plants did not significantly differ from the control while others were significantly different, which is not in agreement with Weng *et al*. [23]. Agronomic characteristics and yield, e.g. stalk length, millable stalks and theoretical sucrose yield, in the medium copy number transgenic sugarcane lines were similar to the non-transgenic control. The theoretical sucrose yields of all 14 transgenic plants were lower than the non-transgenic controls suggesting that Bt protein expression may have influenced the normal sugarcane growth while the theoretical sucrose yield in lines T-3 (51.65 kg), T-8 (50.11 kg) and T-11 (46.68 kg) was not significantly different from the non-transgenic control (55.86 kg).

In conclusion, our results suggest that a medium copy number of *cry1Ac* gene in transgenic sugarcane may be more desirable than too high or too low a number since this appears to compromise gene expression. Higher Cry1Ac protein expression may not be optimum because high protein expression consumes more plant energy and negatively affects agronomic traits. All transgenic lines with medium copy number expressing Cry1Ac protein had relatively equivalent or lower theoretical sucrose yield, compared to controls, and showed significantly improved sugarcane borer resistance. These lines can be potentially used for commercial purposes.

## Supporting Information

S1 Fig Construction of the plant expression vector pGcry1Ac0229. (TIF)

S2 Fig Products of recombinant plasmid pGcry1Ac0229 digested with restriction enzymes. M: DL15,000+2,000 DNA Ladder; 1: The products of pGcry1Ac0229 digested with *Hin*d III and *Eco*R I; 2: The products of pGcry1Ac0229 digested with *Hin*d III.
(TIF)

S3 Fig Partial plantlets in 72-cave trays screened *in vitro* by spraying a solution containing 3.0 ‰ Basta. (TIF)

S4 Fig Real-time fluorescence quantitative standard curve of the *cry1Ac* gene. (TIF)
