# Supplementary figures and images for: Transgenic Sugarcane with a cry1Ac Gene Exhibited Better Phenotypic Traits and Enhanced Resistance against Sugarcane Borer
Source: PLoS One. 2016 Apr 19;11(4):e0153929. doi: 10.1371/journal.pone.0153929 (PMC4836700; doi:10.1371/journal.pone.0153929)

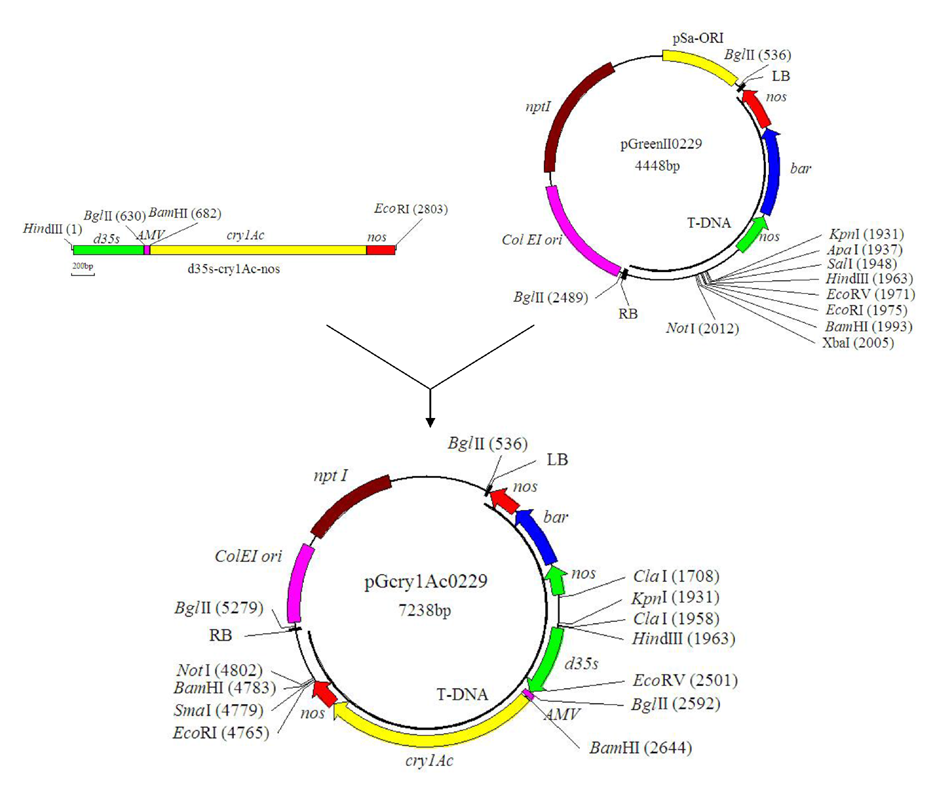

Supplement: S1 Fig — (TIF) [file pone.0153929.s001.tif]

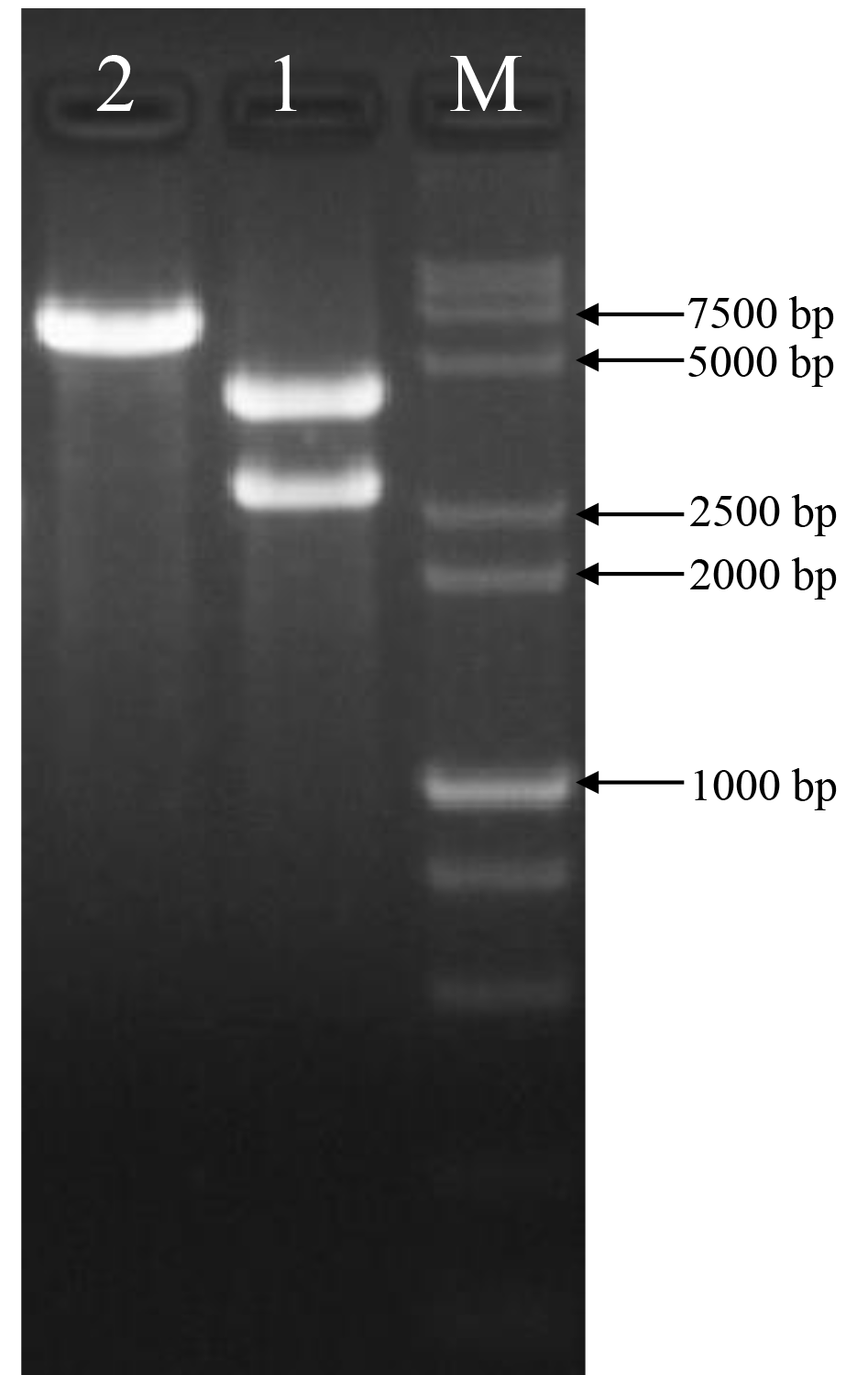

Supplement: S2 Fig — M: DL15,000+2,000 DNA Ladder; 1: The products of pGcry1Ac0229 digested with Hind III and EcoR I; 2: The products of pGcry1Ac0229 digested with Hind III. (TIF) [file pone.0153929.s002.tif]

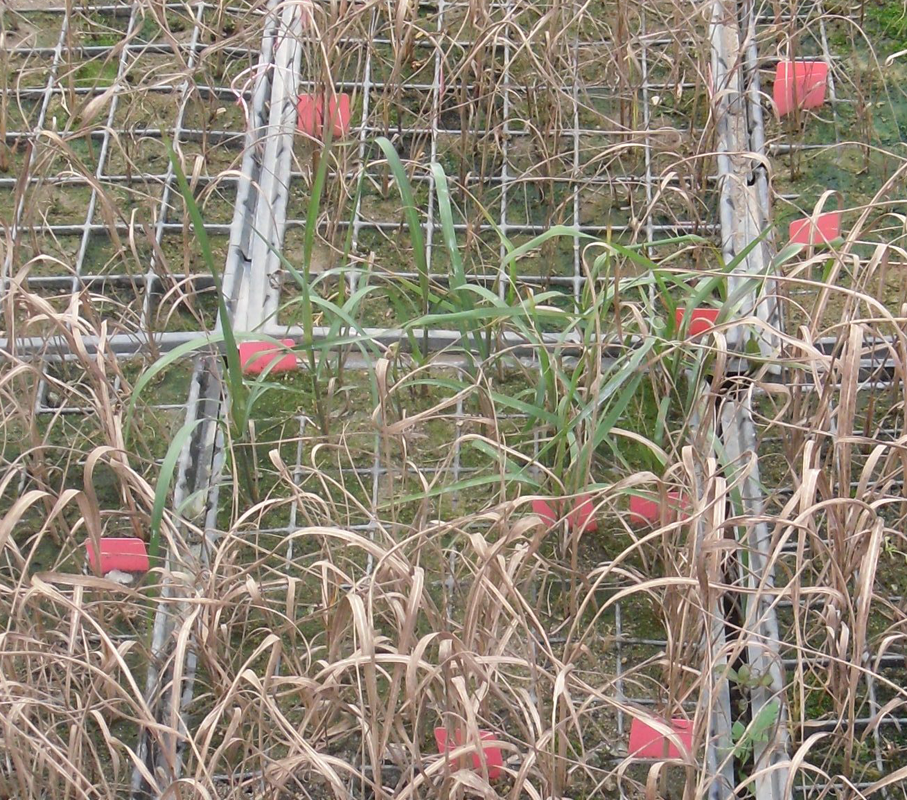

Supplement: S3 Fig — (TIF) [file pone.0153929.s003.tif]

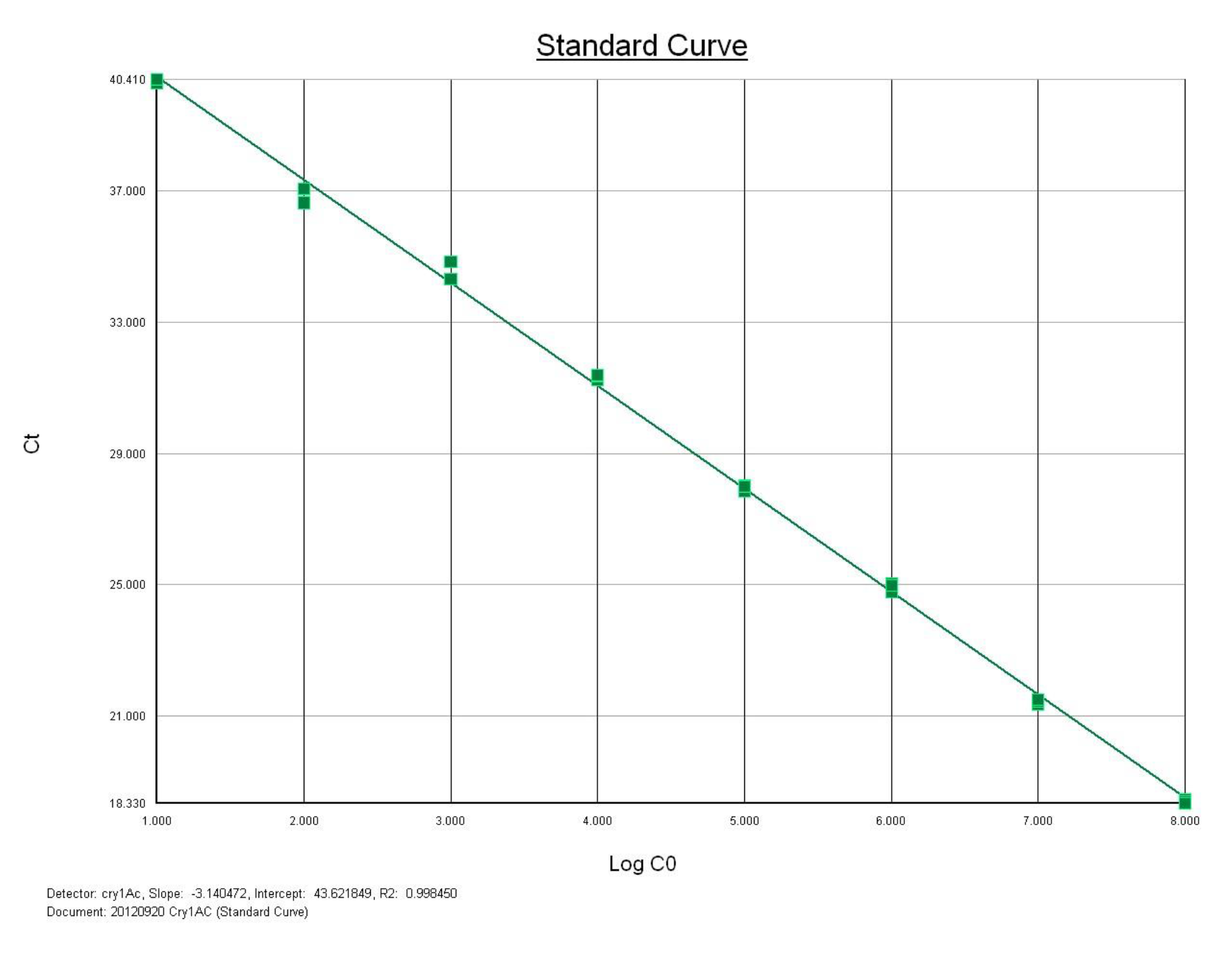

Supplement: S4 Fig — (TIF) [file pone.0153929.s004.tif]
